# The Impact of Physical Activity and Inactivity on Cardiovascular Risk across Women’s Lifespan: An Updated Review

**DOI:** 10.3390/jcm12134347

**Published:** 2023-06-28

**Authors:** Valentina Bucciarelli, Anna Vittoria Mattioli, Susanna Sciomer, Federica Moscucci, Giulia Renda, Sabina Gallina

**Affiliations:** 1 Cardiovascular Sciences Department, Azienda Ospedaliero—Universitaria delle Marche, 60126 Ancona, Italy; valentina.bucciarelli@ospedaliriuniti.marche.it; 2 Department of Medical and Surgical Sciences for Children and Adults, University of Modena and Reggio Emilia, 41124 Modena, Italy; 3 National Institute for Cardiovascular Research-INRC, 40126 Bologna, Italy; 4 Department of Clinical and Internal Medicine, Anesthesiology and Cardiovascular Sciences, University of Rome ‘Sapienza’, Policlinico Umberto I, 49971 Rome, Italy; 5 Department of Neuroscience, Imaging and Clinical Sciences, University of Chieti-Pescara, 66100 Chieti, Italy; giulia.renda@unich.it

**Keywords:** physical inactivity, physical activity, cardiovascular risk, women, gender medicine

## Abstract

Physical inactivity (PI) represents a significant, modifiable risk factor that is more frequent and severe in the female population worldwide for all age groups. The physical activity (PA) gender gap begins early in life and leads to considerable short-term and long-term adverse effects on health outcomes, especially cardiovascular (CV) health. Our review aims to highlight the prevalence and mechanisms of PI across women’s lifespan, describing the beneficial effects of PA in many physiological and pathological clinical scenarios and underlining the need for more awareness and global commitment to promote strategies to bridge the PA gender gap and limit PI in current and future female generations.

## 1. Introduction

Cardiovascular disease (CVD) is the leading cause of morbidity and mortality in the female population [1]. There are substantial gender differences in the pathophysiology of CVD, principally related to estrogen’s protective anti-inflammatory and anti-apoptotic role [2]. Besides traditional CV risk factors, there is an evolving group of risk factors specific to the female gender, including autoimmune disease, breast cancer treatment, cardio-metabolic gestational disorders, and menopause [3,4]. Physical inactivity (PI) is defined as an insufficient physical activity (PA) level to meet present PA recommendations for age and represents a significant modifiable traditional CV risk factor still hard to counteract. This occurs regardless of the abundance of scientific evidence supporting PA as one of the most effective non-pharmacological therapies in primary and secondary CV prevention, with an outstanding effect on vascular homeostasis [5,6,7,8,9,10]. Furthermore, the role of regular PA in preventing and treating non-communicable diseases (NCDs) has been widely demonstrated. The data from a prospective cohort of adults from the United States (63% women) indicated the nearly maximum association with lower mortality achievable by completing, during middle and late adulthood, 150–300 min per week of vigorous PA, 300–600 min per week of moderate PA, or an equivalent combination of both [11,12]. PA showed robust beneficial associations with different mental health conditions, including anxiety and depression, both in the general population and in women, across all lifespans [13,14,15,16,17].

Although the efforts made by the leading international scientific societies to promote adherence to a correct lifestyle, including a healthy diet, adequate levels of PA, and a concomitant reduction in PI, the latest World Health Organization (WHO) records highlighted that, worldwide, 1 in 4 adults and 3 in 4 adolescents (aged 11–17 years) still do not currently meet the global recommendations for PA, with higher levels of PI in economically developed countries [18]. The global costs of PI to healthcare systems are exorbitant, estimated at INT$53.8 billion in 2013 and will reach a cost of INT$520 billion by 2030 if the prevalence of PI does not change [19]. Moreover, globally, PI causes 7.2% of all-cause deaths and 7.6% of CVD deaths, with the more significant relative burden in high-income countries [20]. In women older than 30, the population risk of CVD associated with PI seems to exceed that of other risk factors [21]. The economic burden of PI is disproportionately spread across regions, with the highest economic cost occurring among high-income countries, which account for 70% of expenditure on treatment for illnesses related to PI [22]. On the other hand, a strong association between PA and the risk of developing CVD has been extensively described, with a median risk reduction of CV risk more significant in women than men [23]. Moreover, the level of global CV risk does not alter the inverse connection between PA and incident CVD in women, suggesting that the promotion of PA is essential, regardless of subjective CV risk [24]. Finally, PA in women can be a protective factor in the etiology of many non-traditional CV risk factors, i.e., cardio-metabolic gestational disorders, autoimmune diseases, breast cancer, and breast-cancer-related treatments [25,26,27].

Regardless of the abovementioned outstanding positive effects of PA in women, according to the WHO data PI is more frequent and severe in the female population for all age groups, with a global average of 31.7% for inactive women vs. 23.4% for inactive men [18,28].

Going deeper into the statistical details, the latest National Health Interview Survey (NHIS) data about the levels of PA in the civilian non-institutionalized population of the United States (U.S.) suggested that the prevalence of PI decreased from 40.5% (1998) to 25.6% (2018), with a concomitant increase in meeting the recommended high aerobic PA levels from 26.0% (1998) to 37.4% (2018). However, the prevalence of PI in 2018 was still higher in women (27.8%) than in men (23.2%), and the prevalence of high aerobic PA levels remained lower in women (33%) than in men (42%) [29].

In Europe, about 35.4% of adults, predominantly from southern European countries, were inactive in 2016; in particular, regular PA decreases with age: only 1 in 4 adults older than 55 years old exercises at least once a week. In line with U.S. data, fewer women than men are active in Europe, especially in the youngest age group of 15 to 24 years old (73% of active men compared with 58% of active women) [30].

There are several multifaceted obstacles to women’s participation in PA and sports that can be divided into three main categories: economic and socio-cultural barriers, practical barriers, and knowledge barriers. Among the significant economic and socio-cultural barriers are the wrong belief that sport is masculine and exclusive, low female self-esteem, parents’ disagreement with sport, the fear of scholastic failure, family care, and housework. Practical obstacles include poverty, lack of financial resources, scarcity of leisure time, and scarcity of accessible, safe, and appropriate facilities. Finally, knowledge barriers include the need for more knowledge about the benefits of PA [31,32].

The PA gender gap begins early in life and may have short-term and long-term adverse effects on health outcomes, especially regarding CV status [33].

**Figure 1:**
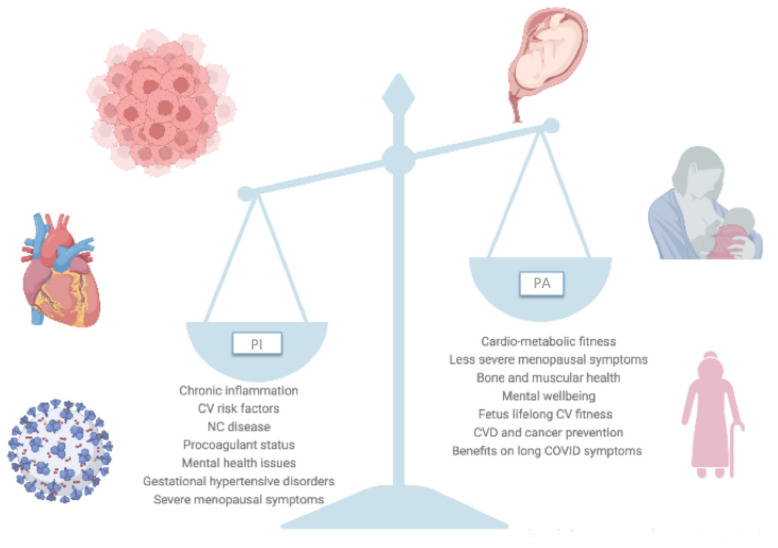
Physical activity (PA) benefits compared with physical inactivity (PI) adverse effects throughout women’s life stages and across different clinical scenarios. Abbreviations: CV, cardiovascular; NC, non-communicable; CVD, cardiovascular disease.

This paper aims to provide an up-to-date review of the evidence of PI and the benefits of PA in the female population throughout all women’s life stages, underlining the need for global commitment to endorse strategies to bridge the PA gender gap, overcome barriers to women’s participation to PA, and limit PI in current and future female generations (Figure 1).

## 2. Physical Activity and Inactivity in Infancy and Adolescence

### 2.1. Benefits of Physical Activity

In children and adolescents, regular PA provides many benefits regarding CV and cardio-metabolic fitness, bone health, mental well-being, and cognitive outcomes. Young people represent 24% of the worldwide population, and investing in their health is crucial, as childhood PA can affect adult health, with a biological and behavioral carry-over effect into adulthood regarding the global health status and a fitter lifestyle [34,35,36].

In 1989, Blair et al. proposed a model for the health consequences of childhood PA, suggesting that three main benefits derive from sufficient childhood PA: 1. improvement in childhood health status; 2. improvement in childhood quality of life; 3. improvement in adult health status. All three could significantly delay the onset of chronic disease and maintain sufficient activity in adulthood [37]. Much scientific evidence supports these hypotheses and confirms PA’s significant positive effect on cardiorespiratory fitness (CRF), body composition, insulin resistance, and CVD risk factors in childhood. Several observational studies documented the dose–response relations between PA and health, suggesting that the higher the PA, the greater the health benefit. However, experimental evidence suggests that even limited amounts of PA, especially if aerobic-based and of moderate or vigorous intensity, can provide great health benefits, especially in high-risk adolescents (i.e., obese with high blood pressure) [38]. It is well known that CRF is a good predictor of CV health starting from childhood, as higher levels of CRF in this period correlate to a better CV profile in adulthood. Data from the Healthy Lifestyle in Europe by Nutrition in Adolescence (HELENA) study, in a population of 3528 adolescents from 10 European centers, confirmed the strong association between CRF and the ideal CV health index, according to the American Heart Association (AHA) indicators, and suggested that a CRF cutoff level of 40–47 mL/kg/min for boys and 35–42 mL/kg/min for girls is associated with a better CV health profile [39]. Other data from the HELENA database demonstrated that vigorous PA, rather than low-intensity PA, is effective in preventing obesity in adolescents, being negatively associated with indices of fat mass and positively associated with markers of muscle mass; in contrast, both average PA and at least moderate PA reduce total and central body fat in youth [40,41]. Moreover, higher vigorous PA (≥30 min/day) and lower sedentary behavior (<2 h/day) have a protective effect on cardio-metabolic risk factors [42]. Another sub-analysis from the HELENA database showed a negative association between PA and markers of insulin resistance, with low CRF modifying this relationship, especially in female adolescents [43]. A systematic review by Janssen et al. examined the relationship between PA and global health in school-aged children and young adults. The authors concluded that even if the results from many observational studies suggest a direct relationship between the amount of PA and the relevance of health benefits, several experimental studies revealed that even a limited volume of PA can have substantial health benefits in the young population at high CV risk. Regarding the type of PA, it seems that aerobic PA is successful at controlling blood pressure within both sexes, even if the effects of the volume and intensity of PA on blood pressure and the effect of age on the relationship between PA and blood pressure are still to be clarified [38]. The beneficial effects of PA on metabolic and psychological status have been confirmed even in children with type 1 diabetes [44]. Furthermore, moderate-to-vigorous PA is also associated with better sleep efficiency, the latter being associated with higher levels of CRF and a more favorable cardio-metabolic profile, as confirmed by a systematic review by Saunders et al. [45,46,47].

### 2.2. Sedentary Behavior and Physical Inactivity Disadvantages

PI in childhood and adolescence is related to unfavorable health adaptations that start from childhood and follow children and adolescents throughout adulthood, leading to higher composite risk factor scores for CVD and a potential decline in CV health [48,49]. PI in children and adolescents leads to increased morbidity since many of the chronic conditions of adults, including early atheromatosis, start in childhood [50].

According to several prospective studies, changes in body fatness are associated with PI; in particular, in children, an inverse relationship between the level of fatness and energy expenditure has been described, suggesting that the latter profoundly impacts the development of obesity [51]. Other adverse health habits have been correlated with PI, such as higher fat intake and cigarette smoking, according to data from the Cardiovascular Risk in Young Finns Study, suggesting that the covariance of PI with other negative health habits in youth affects the development of CVD later in life [52]. Exposure to CV risk factors early in life may influence vascular health, causing modifications to the development of structural and functional vascular changes, i.e., increased intima–media thickness and pulse wave velocity, which are related to early atherosclerosis [53,54]. PI seems to be associated with the accumulation of numerous harmful habits in adulthood, with the strongest association documented in females [52,55]. Consistent with the latest epidemiological data, most adolescents do not meet current PA guidelines, with a trend relatively stable over the past decade [56]. The latest WHO records reported that, in 2018, across 26 European Union Member States, only 17.6% of boys and 9.6% of girls met the recommendation regarding PA, with Portugal, France, and Italy reporting the lowest prevalence of PA among adolescents. An important consideration is that PA prevalence in pediatrics is inversely proportional to age, with the achievement of the recommended amount of daily PA ranging from 24% in children aged 11 to 19% at age 13 and 15% at age 15 [57].

Furthermore, in most countries, girls are less physically active than boys, with a prevalence of recommended levels of PA less than 20% in female adolescents and a subsequent further increase in PI into adulthood [58,59,60,61]. The causes for this gender disparity in PA involvement are still poorly understood [5]. Family support appears to be a consistent factor associated with the PA of both male and female adolescents; in contrast, low self-esteem, lack of interest and awareness about the role of PA, time limitations, scarcity of economic resources, and parental authority seem to influence girls’ participation in PA, especially in low-income countries [62,63]. Ricardo et al. have recently examined records from the Global School-Based Student Health Survey, collected among adolescents from 13 to 17 years old from 64 Global South countries between 2010 and 2020. The pooled ratio for all countries showed that boys presented a PA prevalence 1.58 times higher than that of girls on average, with the highest absolute and relative inequalities in high-income countries [64].

### 2.3. Proposal for Intervention

As stated in the 2020 WHO guidelines, school-age youth (5–17 years) should participate daily in 60 min or more of moderate-to-vigorous PA (MVPA), mostly aerobic; activities focused on musculoskeletal strength should be incorporated at least 3 days a week. Sedentary behavior (SB, defined as any waking behavior characterized by an energy expenditure of ≤1.5 metabolic equivalents while in a sitting, reclining, or lying posture) should be limited as much as possible, especially in terms of recreational screen time [65,66].

The “PI pandemic” should be prevented from early childhood, as there is no doubt that early lifestyle-related factors significantly influence individual’s biological risk factor profile, and childhood appears to be the most appropriate period for positive lifestyle adoption. Schools should offer children curriculums concentrated on the harmful effects of PI and on the positive effects of PA, and should strongly encourage physical practices to ensure that the recommendations for daily PA are embraced and met in student populations and to reduce SB [67]. Physical education teachers should be conscious of their central role in limiting gender inequality, endorsing activities potentially appealing for female students, and eliminating heterosexism and homophobia [68]. According to the latest evidence from systematic reviews, the most successful school-based interventions among adolescents to reduce PI used whole-of-school methodologies combining curricular activities with the broader school environment and the local community [69]. Moreover, playing on sports teams and participating in physical exercise classes may contribute more to global activity in girls [70]. However, these interventions demonstrated only minor results when PA was assessed quantitatively, i.e., using an accelerometer [71,72]. School-based interventions should promote PA programs that institutional teams will be determined to implement and that the involved adolescents are encouraged to support. PA initiatives should focus on the specific requests and necessities of adolescents. In this regard, a study by James et al. explored the recommendations made by a group of teenagers from secondary schools to improve PA engagement, highlighting a significant gap between the most proposed activities and the adolescents’ needs. According to adolescents’ suggestions, the activities should be cheaper, more locally accessible, and specific to teenagers, with a broader choice of proposed activities. Teenage girls stressed their need to engage in enjoyable activities that should not be competitive but friendly and entertaining. Moreover, both boys and girls strongly agree on the need for increased opportunities to participate in more unstructured activities [73]. Finally, it is crucial to offer sufficient education on gender equality for teachers and students, and future research is needed to further clarify the role of all the social and environmental factors potentially related to PI, to propose new approaches to overcome the inactivity phenomenon from the first decades of life.

## 3. Physical Activity and Inactivity in Pre-Pregnancy, Pregnancy, and Post-Pregnancy Period

### 3.1. Benefits of Physical Activity

The AHA statement on women’s CV health underlines the importance of lifestyle interventions in the “Life’s Simple 7”, a list of the 7 most important health factors (diet, PA, nonsmoking, body mass index, blood pressure, lipids, and glycemia), recently revised to “Life’s Essential 8”, incorporating sleep health as the 8th metric [74]. Among the abovementioned CV health metrics, PA can counteract CV risk factors before, during, and after pregnancy, and according to the latest evidence, the improvement in maternal cardio-metabolic health is reflected in the cardio-metabolic health of the fetus and future offspring [75,76]. The exposure of a fetus or neonate to specific risk factors, namely, developmental programming, can influence the development of CVD in later life. Much evidence has confirmed that maternal CV risk factors can influence both endothelial and glucose homeostasis in offspring, increasing the risk of developing early endothelial dysfunction and insulin resistance. In contrast, hypertensive disorders of pregnancy (HDP) can affect maternal health and fetal growth, which are, in turn, associated with increased CV risk later in life [77,78,79]. Moreover, it seems that CV risk factors, both micro- and macro-vascular, track from mother to child, regardless of environmental exposures and pregnancy complications, causing an adverse CV profile in the offspring at a 6-to-9 year follow-up [80].

There are several benefits of PA for maternal cardio-metabolic health including positive vascular remodeling and angiogenesis, improved endothelial function and arterial stiffness, reduced oxidative stress, and decreased levels of inflammatory cytokines and cortisol [81,82]. A greater amount of leisure-time PA in the first trimester of pregnancy leads to a lower risk of adverse pregnancy outcomes (APOs) [83]. Additionally, women who exercise as recommended have a 30% lower risk of developing HDP, including gestational hypertension and pre-eclampsia, and experience a reduced CV risk profile in perimenopause [81,84]. PA has also been associated with a meaningful reduction in gestational weight gain and post-gestational weight retention, both related to a higher risk of short- and long-term CV events, especially in women with a history of gestational diabetes and HDP [85,86,87]. Finally, PA causes a decline in the odds and severity of maternal mental issues, i.e., anxiety and prenatal depression, related to an increased risk of new CVD within 24 months postpartum. Moreover, PA significantly improves the maternal quality of life, along with reduced stress and cortisol levels, both associated with lower maternal oxidative stress and a better long-term metabolic environment of the offspring [82,88,89]. Furthermore, PA improves fertility and assisted reproductive therapy outcomes, as well as metabolic profile in polycystic ovary syndrome, which is recognized as the leading cause of anovulatory infertility [90,91].

### 3.2. Sedentary Behavior and Physical Inactivity Disadvantages

A statement from the American Heart Association (AHA) on women’s CV health and its influence on pregnancy complications has been recently published [92]. According to the latest statistics, less than 1% of young adults of reproductive age have optimal CV health, and almost 1 in 5 births experiences an APO, with a substantial increase in cases over the past decade, especially regarding HDP [93,94].

A recent study by Silva-Jose et al. showed that although in the last 15 years there has been a substantial intensification in physical practice in the pregnant population, the current levels are still very far from the international recommendations [95]. Even if more than two-thirds of pregnant women participate in some type of recreational PA, the percentage of pregnant women exercising at the recommended level is still low, ranging from 15 to 27.3% [96,97]. Recent data from a Swedish epidemiological study showed a correlation between longer sedentary time during pregnancy and the increase in blood loss during delivery/postpartum, as well as worse self-rated health during pregnancy [97]. Pregnancy determines several physiological, cardio-metabolic adaptations in the mother, essential to support fetal development. In women with pre-pregnancy elevated cardio-metabolic risk factors, mainly exacerbated by PI, these phenomena may indicate the occurrence of APOs [98,99]. APOs are strongly related to the risk of subsequent CVD and long-term kidney disease, and the pre-pregnancy period could be involved in the pathophysiology of APOs [98,100]. For example, women with obesity and abnormal pre-pregnancy blood pressure, as well as women with pre-pregnancy insulin resistance or a family history of diabetes, are more likely to develop pre-eclampsia or gestational hypertension or gestational diabetes, respectively, compared with women without these conditions [101,102]. Among APOs, HDP seems to be associated with an increased risk of atherosclerotic CVD, hemorrhagic stroke, and heart failure. In contrast, gestational diabetes, preterm delivery, placental abruption, miscarriages or stillbirths, and the presence of anomalies in the weight of the newborn seem to be associated with an increased risk of atherosclerotic CVD [98]. The association between APOs and the risk of subsequent CVD is so important that the 2011 AHA guidelines for the prevention of CVD in women recommends including a history of APOs in the CVD risk evaluation in women. Moreover, APOs should be considered CV risk enhancers in evaluating statin prescriptions for CVD prevention [103,104]. Additional studies are needed to assess the impact of different levels of sedentary time on pregnancy outcomes [97].

### 3.3. Proposal for Intervention

The 2020 WHO guidelines on PA and SB recommend that all pregnant women, without contraindication, should do at least 150 min of moderate-intensity aerobic PA throughout the week, with a variety of aerobic and muscle-strengthening activities, replacing sedentary time with PA of any intensity, including light intensity [105,106].

Similarly, many governments have developed guidelines for PA during pregnancy, recently summarized in a review by Hayman et al., highlighting the remarkable concordance in the recommendations offered worldwide [107].

All women’s healthcare providers should absorb and adopt the guidelines and efficiently support safe involvement in PA before, during, and after pregnancy, with effective lifestyle counseling that should start in the pre-conceptional period and continue during the postpartum months and beyond, as PA represents an investment in future CV health, especially during the menopausal transition [98,108].

## 4. Physical Activity and Inactivity in Perimenopause and Beyond

### 4.1. Benefits of Physical Activity

Menopause is considered one of the emergent non-modifiable CV risk factors in the female population, being associated with a decline in ovarian hormone concentrations that leads to cardio-metabolic negative adaptations and increased inflammatory status [2,109]. The noticeable changes in cardio-metabolic health observed in this scenario may be partially explained by modifiable lifestyle factors such as PI [110]. In this regard, PA represents a valuable tool to counteract these undesirable adaptations, especially if women exercise with a high level of adherence to a fitness program. Moreover, PA can improve the immune-neuroendocrine profile and serum angiogenic properties during the menopausal transition [111,112,113,114,115]. Regarding aerobic exercise, continuous aerobic training and high-intensity aerobic interval training can elicit the same physiological benefits in terms of a reduction in plasma glucose, insulin, homeostasis model assessment-adiponectin, and insulin resistance and an increase in plasma high-density lipoprotein-cholesterol, adiponectin, and aerobic fitness [116]. Perimenopausal women can also benefit from regular strength training, which can help to improve bone density, reduce body fat, and build skeletal muscle mass, maintaining adequate physical performance [117,118,119]. Both aerobic and resistance training, alone or in combination, can improve CRF and muscular strength in this population [120]. Moreover, a moderate-to-intense PA is crucial to protect or ameliorate cognitive health through body movement [121]. Finally, a recent study by Wu et al. suggests a strong negative correlation between PA and the severity of menopausal symptoms, with higher PA levels correlated with a better perceived health status [122].

### 4.2. Sedentary Behavior and Physical Inactivity Disadvantages

Despite the abovementioned and well-known health benefits, few adults, and fewer older adults, especially in the postmenopausal female population, meet recommended guidelines [123]. Furthermore, older women seem generally more sedentary and less active than older men [124].

In this population, SB has been associated with metabolic disorders, obesity, CVD, cancer, mortality, and psychological distress, as well as with adverse changes in coagulation homeostasis and severe menopausal symptoms [125,126]. Therefore, reducing sedentary activity provides an alternative strategy to reduce the risk of CVD and CVD-related mortality [108,127,128]. Further attention should be paid to supporting menopausal women in maintaining an adequate level of spontaneous PA when they regularly exercise, as it seems that the involvement in a planned program of physical exercise may result in a decline in spontaneous PA, which in turn reduces the positive effects of exercise on lipid profile [129,130]. The baseline spontaneous PA and leptin-to-fat-mass ratio of postmenopausal women involved in exercise training seem to be negative and independently correlated with a subsequent reduction in spontaneous PA [131].

### 4.3. Proposal for Intervention

The 2020 WHO guidelines on PA and SB recommend that adults and older adults with chronic conditions should perform at least 150–300 min of moderate-intensity aerobic PA, or at least 75–150 min of vigorous-intensity aerobic PA, or an equivalent combination of moderate- and vigorous-intensity activity throughout the week for substantial health benefits [105].

Every woman needs to find an enjoyable activity that fits into her daily lifestyle [132,133]. Menopausal women should aim for 30 min of moderate-intensity PA every day, i.e., walking, jogging, swimming, cycling, dancing, and gardening. Other beneficial activities include strength training and balance exercises, which are especially important as women age which can increase the risk of falls. Healthcare professionals should actively promote PA as a cheap and effective therapy free of side effects in menopausal women, taking into account both known facilitators (i.e., program adaption, gratification, and setting) and strategies to overcome barriers to PA participation (i.e., lack of social and economic support and exercise experience) in order to improve women’s adherence to fitness programs [134,135]. In this sense, the positive effects of PA should be optimized according to women’s life habits. For instance, the evening execution of a walking program may lead to better positive effects in terms of body composition improvement, potentially linked to spontaneous dietary habit modification [136]. Increased sedentary time should also be strongly discouraged as a negative compensatory adaptive response to exercise training [131].

## 5. Physical Activity and Inactivity in CVD

### 5.1. Benefits of Physical Activity

The inverse association between PA and CVD has been extensively validated, especially in high-risk subgroups, including patients with metabolic syndrome, current smokers, and older adults.

This positive relationship has also been confirmed in the female population, as shown by Paynter et al. in the WHI-OS (Women’s Health Initiative Observational Study), demonstrating that recreational PA was the only lifestyle factor independently associated with incident CVD when added to traditional risk factor models [137]. PA seems to be similarly effective in preventing CVD among women with varying levels of global CV risk [24]. Even light-to-moderate PA is associated with lower coronary heart disease rates in women, and higher daily life movement has been independently associated with a lower CV risk in older women [138]. Combining different PA interventions is the most effective way to reduce CV risk factors in women [139].

### 5.2. Sedentary Behavior and Physical Inactivity Disadvantages

Current records from clinical trials suggest that PA alone is not enough to reduce the risk of CVD, especially in older adults, as both PI and SB negatively influence CV health status, especially in older women, regardless of the level and intensity of PA [127]. Data from the Women’s Health Initiative confirmed the presence of a linear connection between more significant amounts of sedentary time and mortality risk after controlling for multiple potential confounders [140]. In the same population, prolonged sitting time was associated with increased CVD risk in postmenopausal women without a history of CVD, independent of leisure-time PA [141].

Moreover, Ekelund et al. described a statistically significant higher risk of death for sedentary times of 9.5 or more hours daily [127,142,143]. On the other hand, lower sedentary time is associated with lower all-cause mortality [144]. High sedentary time and long mean bout duration have been associated in a dose–response manner with increased CV risk in a subcohort from Women’s Health Initiative [126]. Moreover, a positive correlation between prolonged SB periods and worsening arterial stiffness, a well-known prognostic marker for CVD, has been recently highlighted in a population of 1125 women from the Physical Activity and Health in Older Women Study, with more prolonged bouts of SB being associated with higher levels of arterial stiffness [145]. Patients with CVD exhibit considerably higher amounts of SB than healthy controls and show low engagement in moderate-to-vigorous PA even following specific cardiac rehabilitation programs [146].

Duran et al. demonstrated, in a population of 149 patients with acute coronary syndrome (30.2% women), that during the first month post-discharge there is a significant tendency to accumulate high volumes of sedentary time, with most patients showing slight improvement over time [147]. This negative lifestyle adaptation is associated with a worse long-term prognosis among patients with acute coronary syndrome as high SB, mainly when associated with low PA, strongly correlates with poor cardiorespiratory fitness [148]. Incremental tertiles of time-varying SB also correlate with an increased risk of incident HF in postmenopausal women, according to the data from the Women’s Health Initiative Observational Study by LaMonte et al. [149].

### 5.3. Proposal for Intervention

The latest guidelines on CVD prevention in clinical practice, endorsed by the European Society of Cardiology, suggest that every patient with atherosclerotic CVD events or with a history of heart failure should participate in a medically supervised, structured, and exercise-based cardiac rehabilitation program that should start as soon as possible after the initial CV event. The program should be tailored to each patient and include both aerobic and resistance exercises [150,151,152]. A specific tool, namely, the EXPERT tool (Exercise Prescription in Everyday Practice and Rehabilitation Training), has been proposed to optimize exercise training. Home-based telemonitoring and telehealth interventions have been suggested to increase rates of participation. The efforts of every clinician should be focused on the improvement of adherence to a rehabilitation program and on specific interventions aimed at reducing SB, i.e., the use of an interactive accelerometer equipped with cloud-based services to store and monitor patient’s habitual activity online in order to create a patient’s habitual activity and SB profile, which can be supervised over long periods of time [152,153,154,155].

## 6. Current Evidence on Physical Activity and Inactivity in the COVID-19 Pandemic

It has been widely demonstrated that the health policy reactions to the COVID-19 pandemic, with lockdown and significant movement restrictions, caused widespread effects on CV risk. The significant limitation of economic and social activities has led to unemployment, increased sedentary time, social isolation, and increased incidence of mental health issues, all of which are well-recognized risk factors for CVD and associated with worsening CV outcomes [156]. The pandemic has been a stressful time for everyone, especially for women when juggling work and home life. Women are often expected to be the primary caretakers for their families, and with the extra stress of the pandemic, it has been even more difficult for them to manage.

Many studies demonstrated that women of all ages were significantly less physically active than men during COVID-19 and reported more barriers and fewer facilitators to PA than men, with a significant worsening in psychological health. On the other hand, women who engaged in more PA had improved mental health scores [157,158,159,160].

Given these premises, home-based PA programs for the prevention of PI and SB during the COVID-19 era have been strongly suggested as powerful tools to preserve both general and CV well-being and mental health, especially in women who were severely affected by emotional stress and anxiety, with a potentially devastating impact on CV risk burden [161,162,163,164,165,166,167].

Recently, the “Long COVID syndrome” or “post-acute sequelae of COVID-19” (PASC) is emerging in clinical practice. This condition occurs 3 or more weeks after the original infection and is characterized by symptoms lasting for at least 2 months, with no other explanation, in subjects who have had a severe, moderate, or mild form of COVID-19, mainly females [168,169,170,171,172]. The persistence of SARS-CoV-2 symptoms severely affects functional and emotional status, as well as leisure-time PA, especially in the female population, limiting PA participation and decreasing both CV health and quality of life [173,174,175].

PASC has been associated with more than 100 symptoms, including fatigue, anxiety, depression, sleep disorders, and CV symptoms and complications, including palpitations, chest pain, and dyspnea, the latter being reported in 5–29% of COVID-19 survivors [176]. Furthermore, independent of symptom burden, women with PASC seem to experience a worsening in vascular health, with higher levels of blood pressure and central arterial stiffness [177]. The latest consensus statement by the American Academy of Physical Medicine and Rehabilitation highlighted that PI is strongly correlated with CV morbidity and mortality, more severe COVID-19, and risk of PASC [178]. The authors recommend exercise training as an effective intervention to improve both mental health issues and CV complications, paying attention to minimizing or avoiding post-exertional symptom exacerbation (PESE), which has been extensively described in this population [179,180,181,182,183].

## 7. Conclusions and Future Directions

PI represents a real global emergency as it significantly affects general and CV well-being, especially in women that are globally more inactive compared with men. Gender differences in terms of PA are tangible and exist across all age groups and clinical scenarios. The equal opportunity for everyone to be active from a young age and maintain activity should be provided worldwide and would represent an actual investment in short- and long-term global health.

Many studies confirmed that increasing PA in the population would reduce working-age mortality and morbidity and increase productivity, with significant economic gains for the economy worldwide, especially in high-income countries [184]. Moreover, pre-pregnancy and pregnancy CV health should be considered a central target to improve women’s lifelong health but also the health of the birthing individuals over their life course [92]. Recently, the latest global status report on PA by the WHO highlighted that, although national guidelines to fight NCDs and PI have increased in recent years, currently only 72% of policies are reported to be supported or applied. Moreover, it seems that only just over 50% of countries have planned a mass participation PA event or a national communications campaign about PA in the last 2 years. Governments should help break down barriers to women’s participation in sport, promoting different regulations to provide everyone with access to PA and suitable infrastructures to ease protected access and privacy in facilities. Schools and universities, sports societies, non-governmental associations, and local initiatives can also play an essential role in accelerating this revolution, spreading the need for gender equality in PA and promoting projects focused on the existing barriers to women’s awareness of and access to PA, and claiming more space and involvement for women in sport. Proposed activities should be tailored to the specific requests and necessities of the female population of every age.

Health education messages supplied to mobile devices, focused on the role of an active lifestyle on CV fitness, promoting the WHO’s guidelines on PA levels, and explaining the adverse effects of PI and SB by captivating visual content may constitute an effective tool to improve health literacy, especially in the youngest population [185].

Strategies to reduce the gender gap should be highlighted in efforts to increase PA levels in all age groups and in all countries, from childhood to old age, to achieve radical changes at every level through multidisciplinary and cross-sectoral collaboration to increase levels of PA in current and future generations, as stated in the WHO Global Action Plan on PA 2018–2030 [64,186].

## Data Availability

Not applicable.
